# Supplementary material for: Free fatty-acid transport via CD36 drives β-oxidation-mediated hematopoietic stem cell response to infection
Source: Nat Commun. 2021 Dec 8;12:7130. doi: 10.1038/s41467-021-27460-9 (PMC8655073; doi:10.1038/s41467-021-27460-9)
Supplement: Supplementary file 1 — Supplementary Information [file 41467_2021_27460_MOESM1_ESM.pdf]

Supplemental Information

Free fatty acid transport via CD36 drives β-oxidation mediated hematopoietic stem cell response to infection

Jayna J Mistry[a,b], Charlotte Hellmich[a,c], Jamie A Moore[a], Aisha Jibril[a], Iain Macaulay[b], Mar Moreno-Gonzalez[d], Federica Di Palma[a,b], Naiara Beraza[d**], Kristian M Bowles[a,c**] and Stuart A Rushworth[a**]

[a] Norwich Medical School, University of East Anglia, Norwich Research Park, Norwich, NR4 7UQ, United Kingdom.

[b] Earlham Institute, Norwich Research Park, Norwich, NR4 7UH, United Kingdom

[c] Department of Haematology, Norfolk and Norwich University Hospitals NHS Trust, Colney Lane, Norwich, NR4 7UY, United Kingdom

[d] Gut Microbes and Health Institute Strategic Programme, Quadram Institute, Norwich, United Kingdom

Supplementary figure 1

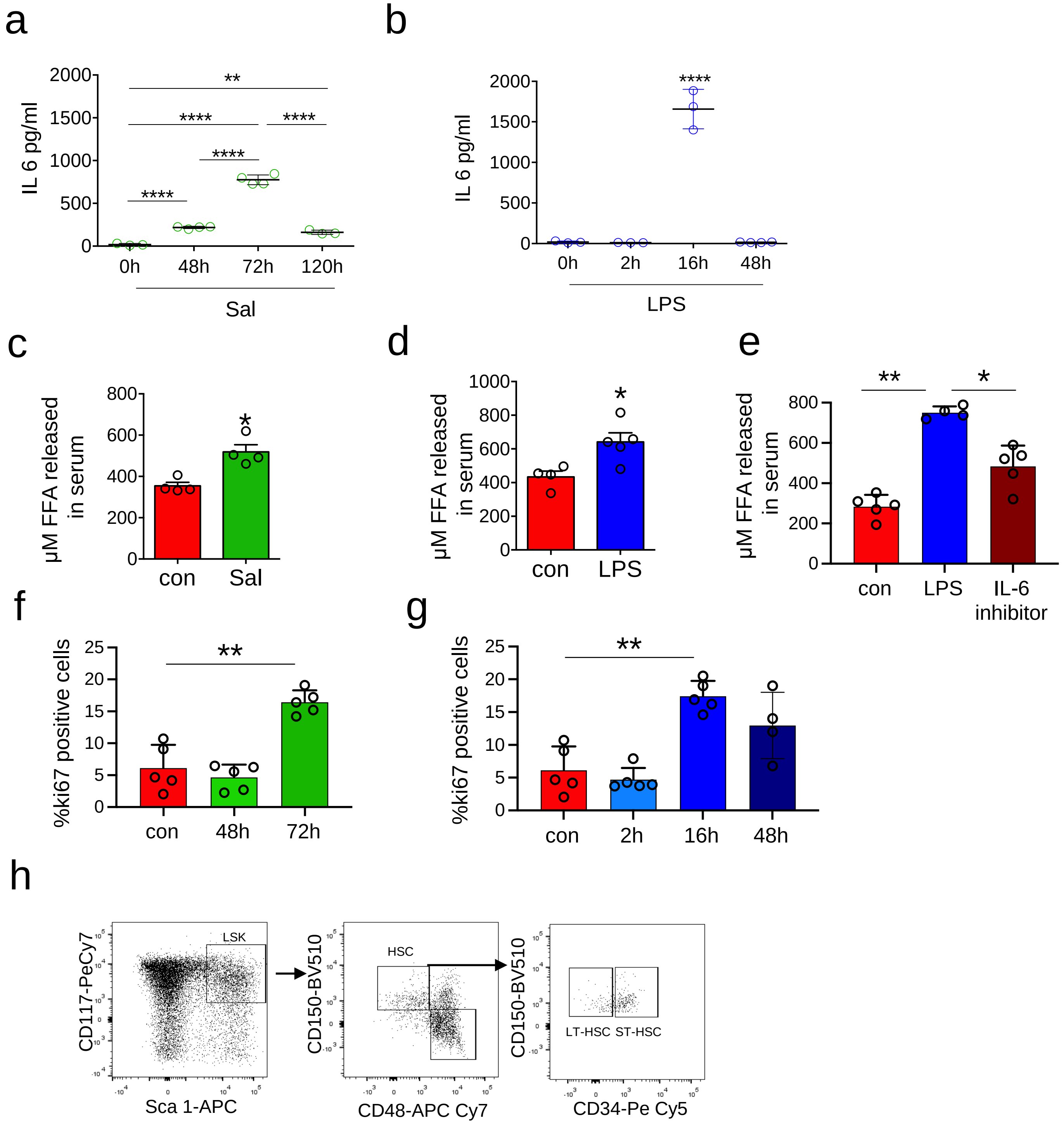

Supplementary Figure 1. IL-6 and free fatty acid release in response to infection. C57BL/6 mice were infected with (A) *S.typhimurium* (Sal) for 48, 72 and 120 hours (A) or LPS for 2, 16 and 48 hours (B). Blood was taken from the animals by cardiac puncture and the serum was assessed for levels of IL-6 by ELISA. C57BL/6 mice were infected with (C) *S.typhimurium* (Sal) for 72 hours or (D) LPS for 16 hours. Blood was taken from the animals by cardiac puncture and the serum was assessed for levels of FFA. n=4 (E) C57BL/6 mice were treated with IL-6 inhibitor (clone MP5-20F3) followed by LPS for 16 hours. Blood was taken from the animals by cardiac puncture and the serum was assessed for levels of FFA. C57BL/6 mice were infected with (F) *S.typhimurium* (Sal) for 48 or 72 hours (G) or LPS for 2, 16 and 48 hours. Percentage of cycling cells as measured by Ki67 positive cells. n=5 in each group. (H) The gating strategy to analyse LSK and HSC population of LPS treated mice is shown. The Mann-Whitney U test (two-tailed) was used to compare between treatment groups *p<0.05 **p<0.01.

Supplementary figure 2

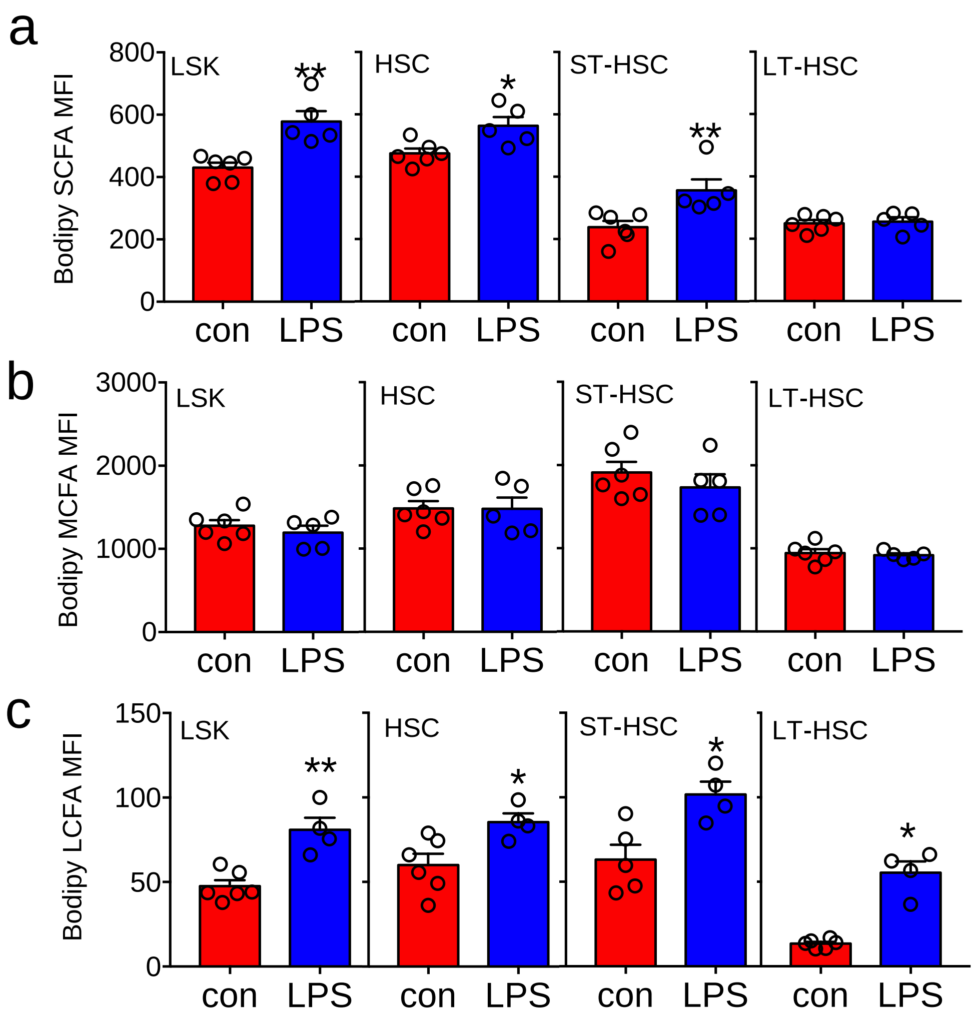

Supplementary Figure 2. FFA uptake in HSPC in response to LPS. C57BL/6 mice were treated with 1mg/kg LPS for 16 hours. (a) The mice were sacrificed and the bone marrow was extracted and stained with BODIPY C5 (BODIPY™ FL C_5-Ceramide (*N*-(4,4-Difluoro-5,7-Dimethyl-4-Bora-3a,4a-Diaza-*s*-Indacene-3-Pentanoyl)Sphingosine)) representing accumulation of short chain fatty acids (SCFA) n>5 in each group. (b) C57BL/6 mice were treated with 1mg/kg LPS for 16 hours. The mice were sacrificed and the bone marrow was extracted and stained with BODIPY C12 (BODIPY™ FL C_{12} (4,4-Difluoro-5,7-Dimethyl-4-Bora-3a,4a-Diaza-*s*-Indacene-3-Dodecanoic Acid) representing accumulation of medium chain fatty acids (MCFA) n>5 in each group. (c) C57BL/6 mice were treated with 1mg/kg LPS for 16 hours. The mice were sacrificed and the bone marrow was extracted and stained with BODIPY C16 (BODIPY™ FL C_{16} (4,4-Difluoro-5,7-Dimethyl-4-Bora-3a,4a-Diaza-*s*-Indacene-3-Hexadecanoic Acid)) representing accumulation of long chain fatty acids (LCFA). N=5 in each group. Data shown are mean ±SD *P<0.05 **P<0.01 The Mann-Whitney U test (two-tailed) was used to compare between treatment groups.

Supplementary figure 3

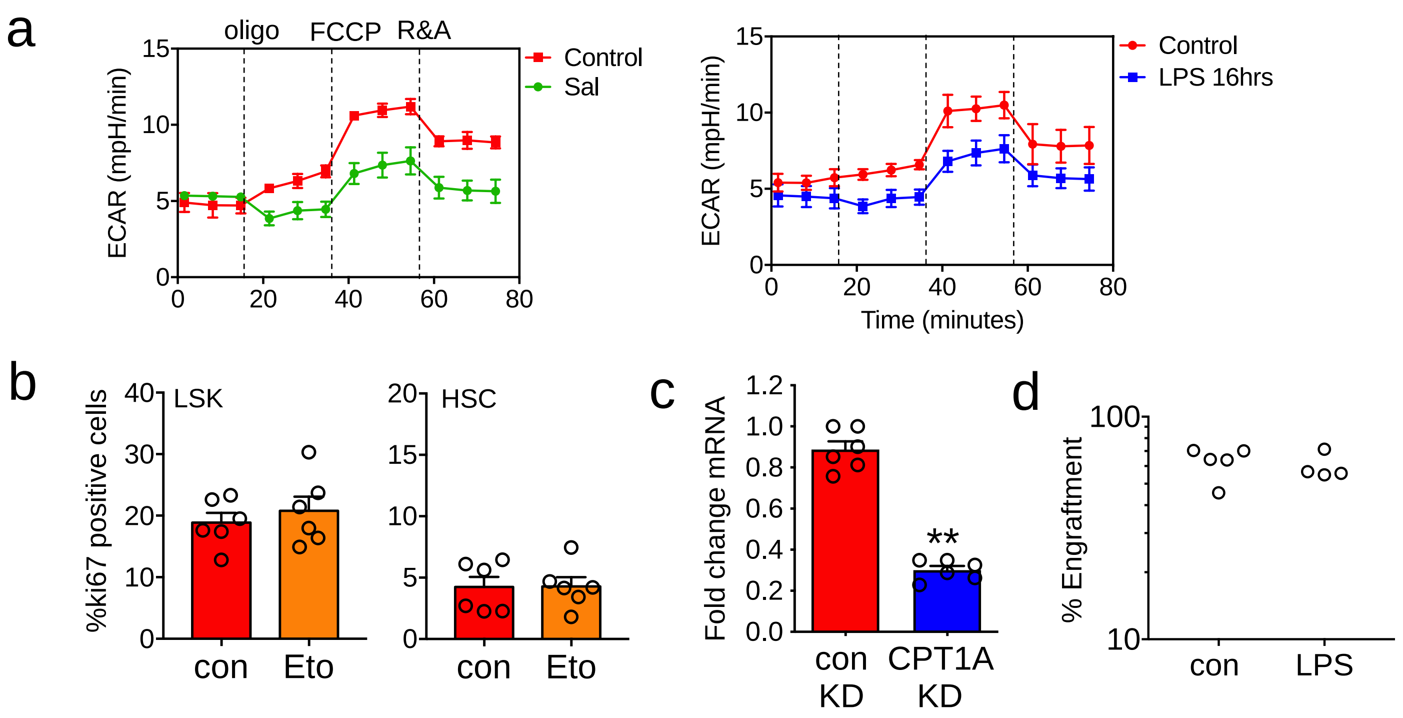

Supplementary Figure 3. LPS and *S.typhimurium* cause a decrease in extracellular acidification rate in LSK. (A) C57BL/6J mice were infected with *S.typhimurium* (Sal) for 72 hours or LPS for 2 hours, the animals were sacrificed, the bone marrow was extracted and the LSK population was isolated by FACS. Glycolysis levels in the LSKs were measured by extracellular acidification rate (ECAR). n=5 mice. (B) C57BL/6J mice were treated with control PBS or 10mg/kg Etomoxir (Eto) for 16 hours. Percentage of cycling cells as measured by Ki67 positive cells. n=6 in each group. (C) Relative CPT1A expression in control knockdown virus (conKD) or CPT1A knockdown virus (CPT1AKD) in CD45.1 lineage negative, CD117 positive (LK) cells. N=5 mice in each group. (D) WT CD45.1 lineage negative, CD117 positive (LK) cells were transduced with a CPT1A knockdown lentivirus ($LK^{CPT1A\ KD}$) was transplanted into WT CD45.2 animals. Post engraftment mice were treated with 1mg/kg LPS for 16 hours and the bone marrow cells were analysed by flow cytometry for CD45.1 expression to confirm engraftment. Data shown are means ± SD of >4 mice in each group. **P<0.01 by Mann-Whitney U test. The Mann-Whitney U test (two-tailed) was used to compare between treatment groups.

Supplementary figure 4

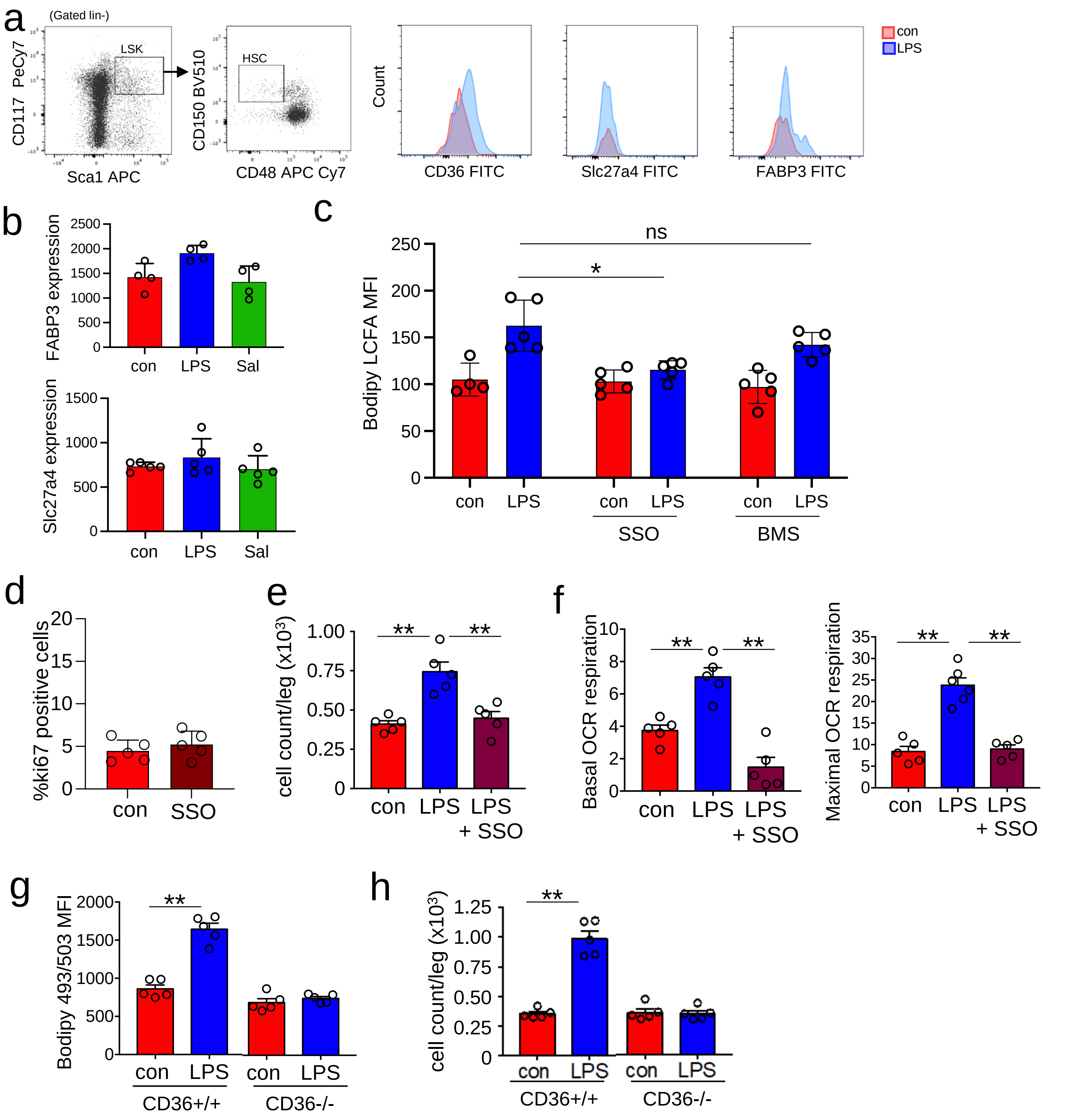

Supplementary Figure 4. CD36 but not Slc27a4, FABP3 regulate LCFA uptake in response to LPS. (A and B) Slc27a4, FABP3 and CD36 expression in the HSC following 1mg/kg LPS for 16 hours or *S.typhimurium* (Sal). The mice were sacrificed, and the bone marrow was extracted and stained. The gating strategy to isolate the LSK and HSC population is shown. N=4 in each group. (C) C57BL/6J mice were treated with control PBS or 1mg/kg LPS for 16 hours. The mice were sacrificed and the bone marrow was extracted and $5x10^6$ cells were incubated with 10uM sulfosuccinimidyl oleate (SSO) or 10uM BMS309403 for 15 minutes before treatment BODIPY C16 (BODIPY™ FL C_{16} (4,4-Difluoro-5,7-Dimethyl-4-Bora-3a,4a-Diaza-*s*-Indacene-3-Hexadecanoic Acid)). Cells were incubated for 4 hours at 37C and then HSC were analysed for accumulation of BODIPY C16 long chain fatty acids (LCFA). N=4 in each group. (D) C57BL/6 mice were treated PBS or with 40mg/kg sulfosuccinimidyl oleate (SSO). Percentage of HSC cycling cells as measured by Ki67 positive cells were measured. n=6 in each group. (E) C57BL/6 mice were pre-treated with 40mg/kg sulfosuccinimidyl oleate (SSO) for one hour before treatment with 1mg/kg LPS for 16 hours. The Mann-Whitney U test (two-tailed) was used to compare between treatment groups $*p<0.05$ $**p<0.01$.

The bone marrow cells were analysed by flow for Bodipy 493/503 mean fluorescence intensity (MFI) in the HSC. n=5 in each group. Number of HSC per 100 000 bone marrow cells. n=5 in each group. (F) The LSK population was isolated by FACS and oxygen consumption rate (OCR) levels were measured by extracellular flux assay. Basal (normalised to rotenone) and maximal mitochondrial respiration of LSK cells from control, LPS or LPS and SSO treated animals. (G) CD36-/- or WT (CD36+/+) mice were treated with 1mg/kg LPS for 16 hours. Lipid content (Bodipy 493/503 mean fluorescence intensity (MFI)) in the HSC population n=5 in each group. (H) Number of HSCs per leg. n=5 in each group. Data shown are means ± SD **P < 0.01 Comparison of two groups was performed using the Mann-Whitney U test. For comparison of more than two groups, Kruskal-Wallis statistical test followed by Dunn's multiple comparisons was used.

Supplementary figure 5

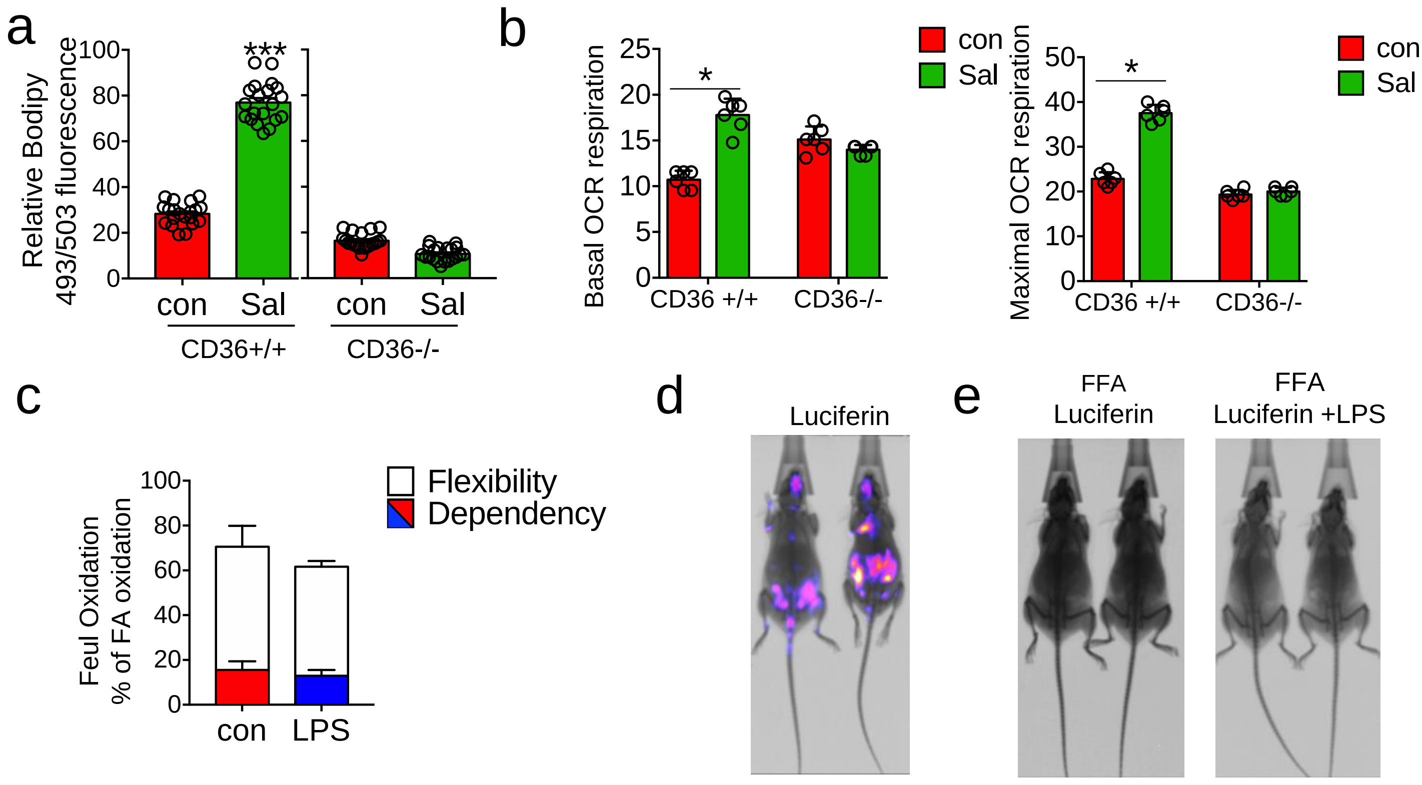

Supplementary Figure 5. CD36-/- HSC have reduced FFA uptake and OCR in response to *S.typhimurium* (A) $CD36^{-/-}$ or WT ($CD36^{+/+}$) mice were infected with *S.typhimurium* (Sal) for 72 hours. Quantification of Bodipy 493/503 fluorescence from live cell fluorescent microscopy in isolated LK cells, 20 LK cells from each mouse in each condition. N=4 in each group. (B) The LSK population was isolated by FACS and oxygen consumption rate (OCR) levels were measured by extracellular flux assay. Basal (normalised to rotenone) and maximal mitochondrial respiration of LSK cells from control or Sal (72 hours) treated $CD36^{-/-}$ or WT ($CD36^{+/+}$) mice. n=5 in each group. (C) $CD36^{-/-}$ were treated with 1mg/kg LPS for 16 hours, the LSK population was isolated by FACS. The LSK population was analysed by seahorse mitochondrial fuel flex kit for the reliance on long chain fatty acids to maintain baseline respiration. n=5 in each group. (D) $CD36^{-/-}$ CD45.2 lineage negative, CD117 positive (LK) cells were isolated and transduced with a firefly luciferase virus (LK^{+FF}) and transplanted into WT ($CD36^{+/+}$) CD45.1 animals. Mice were bioluminescence imaged using luciferin to confirm engraftment. (E) Mice were injected with control PBS for 16 hours then treated with FFA-SS-luc and imaged using bioluminescence (FFA-luciferin). One-week later mice were injected LPS for 16 hours then treated with FFA-SS-luc and imaged using bioluminescence (FFA-luciferin+LPS). Representative images of control and LPS treated mice. n=4 in each group. Data shown are means ± SD The Mann-Whitney U test (two-tailed) was used to compare between treatment groups $^{*}p<0.05$ $^{**}p<0.01$.

Supplementary figure 6

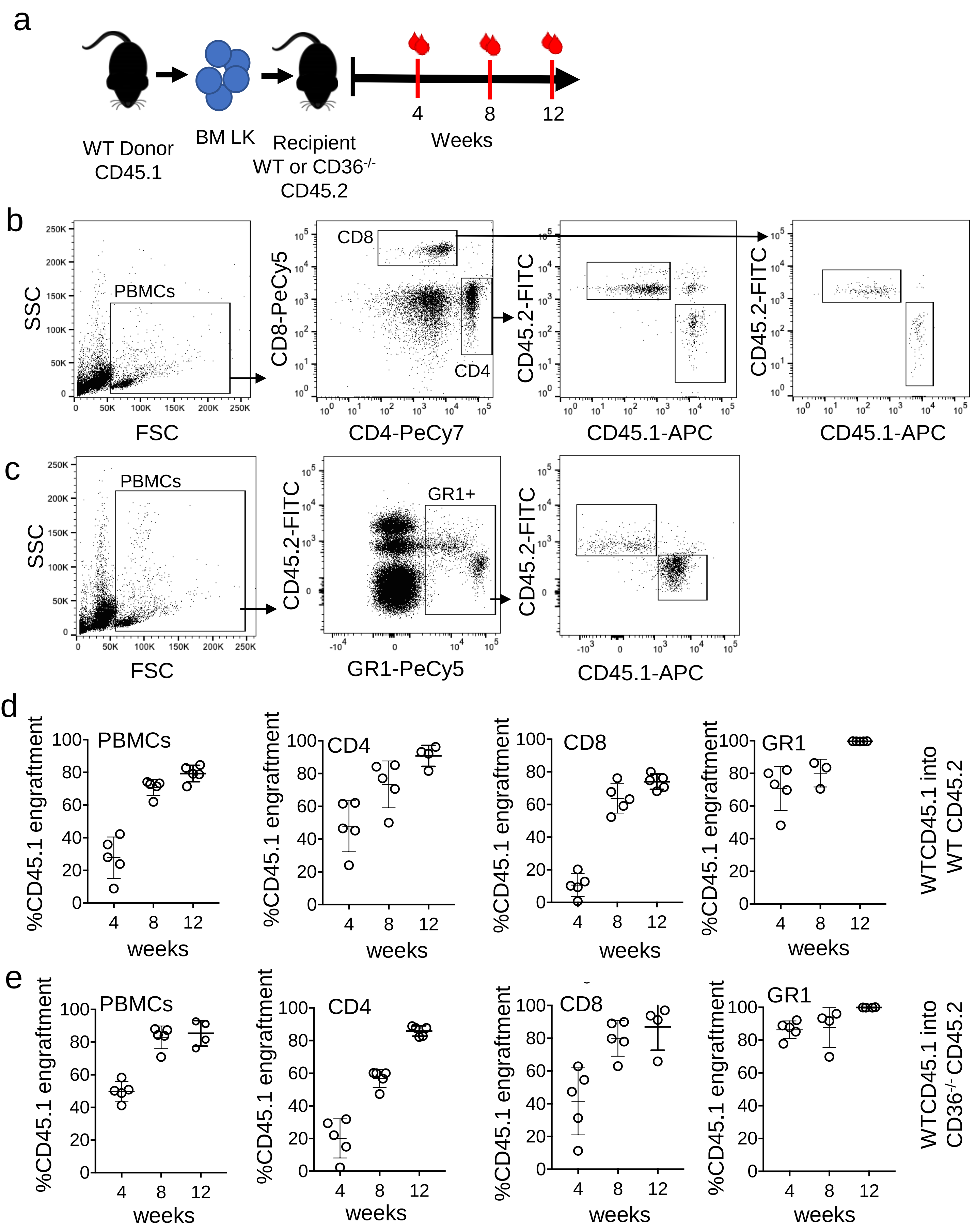

Supplementary Figure 6. Transplantation of WT donor LK cells into CD36-/- animals. (A) C57BL/6 (CD45.2) or $CD36^{-/-}$ (CD45.1) mice were treated with 25 mg/kg busulfan for 3 days prior to tail-vein injections of PepCboy (CD45.1) lineage negative, CD117 positive (LK) donor cells. The peripheral blood was monitored at week 4, 8 and 12 after transplantation and assessed by flow cytometry. (B) The gating strategy for CD4 or CD8 expressing cells is shown. (C) The gating strategy for GR1+ cells is shown. (D) Engraftment was determined by percentage of CD45.1-APC expression on the lymphocytes in the peripheral blood (PB) detected by flow cytometry analysis. Percentage of CD45.1-FITC expressing lymphocytes in the PB of the transplant C57BL/6 mice, CD4-PeCy7 positive population, CD8-PeCy5 positive population and GR1-PeCy5 positive population. N=5 in each group. (E) Percentage of CD45.1-FITC expressing lymphocytes in the PB of the transplant $CD36^{-/-}$ mice, CD4-PeCy7 positive population, CD8-PeCy5 positive population and GR1-PeCy5 positive population. N=4 in each group.

Supplementary figure 7

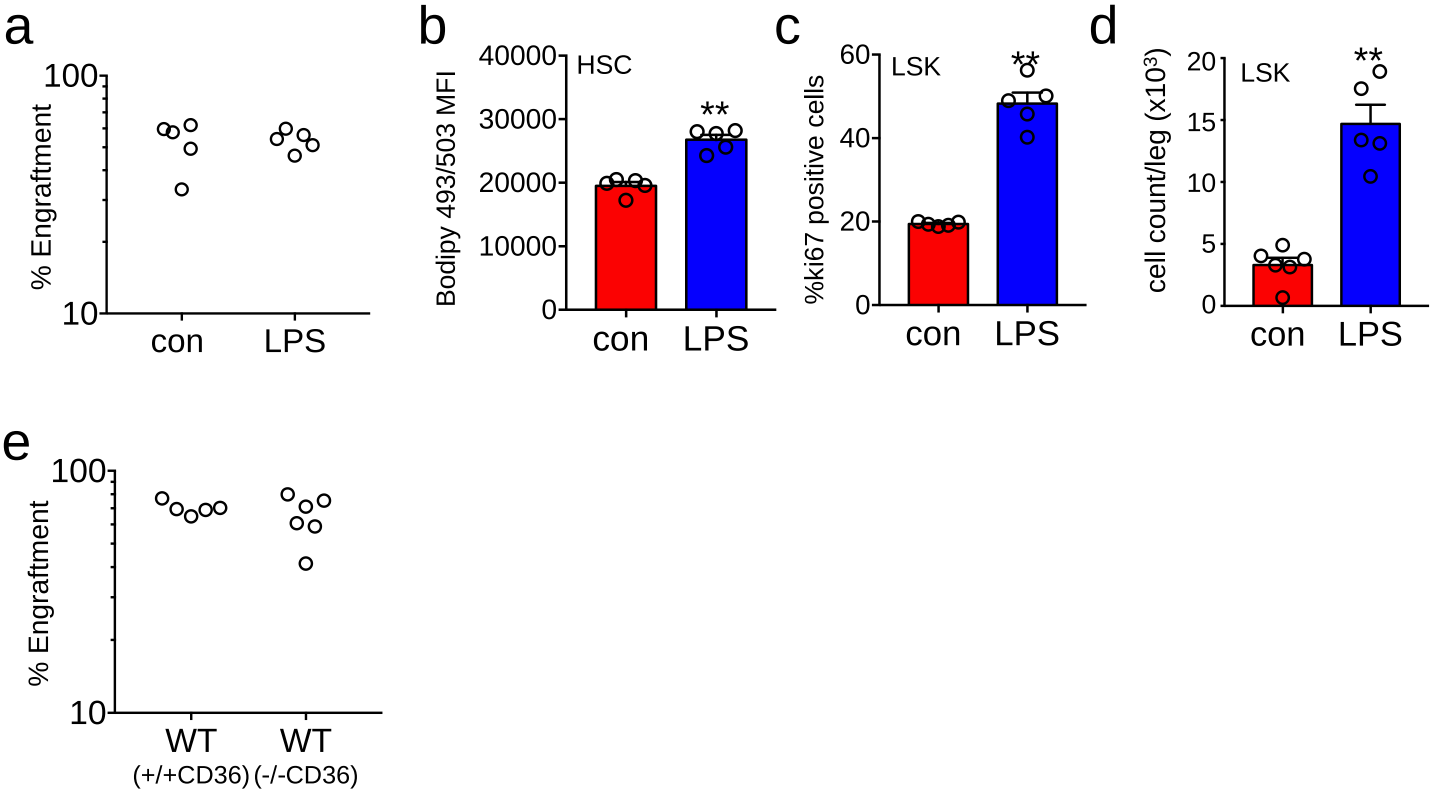

Supplementary Figure 7. Engraftment and FFA uptake of CD36$^{+/+}$ **LK cells into CD36^{-}/- animals** (A) CD36$^{+/+}$ CD45.1 lineage negative, CD117 positive cells were isolated and transplanted into CD36$^{-/-}$ CD45.2 animals. Post engraftment mice were treated with 1mg/kg LPS for 16 hours and cells were analysed by low cytometry. The animals were sacrificed, and the bone marrow was extracted to assess engraftment. n>5 in each group. (B) The bone marrow was stained with a panel of antibodies and Bodipy 493/503 to analyse lipid content (Bodipy 493/503 mean fluorescence intensity (MFI)) in the HSC. N=5 in each group. (C) The bone marrow was stained with a panel of HSC antibodies and Ki67 to analyse cell cycling in the LSK, population. Percentage of cycling cells as measured by Ki67 positive cells after 16 hours LPS treatment. N=5 in each group. (D) Number of LSKs per 100 000 bone marrow cells. N=5 in each group. (E) CD36$^{+/+}$ CD45.2 or CD36$^{-/-}$ CD45.2 lineage negative, CD117 positive cells were isolated and transplanted into WT CD45.1 animals these were termed WT$^{(+/+CD36)}$ or WT$^{(-/-CD36)}$. The animals were sacrificed, and the bone marrow was extracted to assess engraftment. N=5 in each group. Data shown are means ± SD. The Mann-Whitney U test (two-tailed) was used to compare between treatment groups *p<0.05 **p<0.01.

Supplementary figure 8

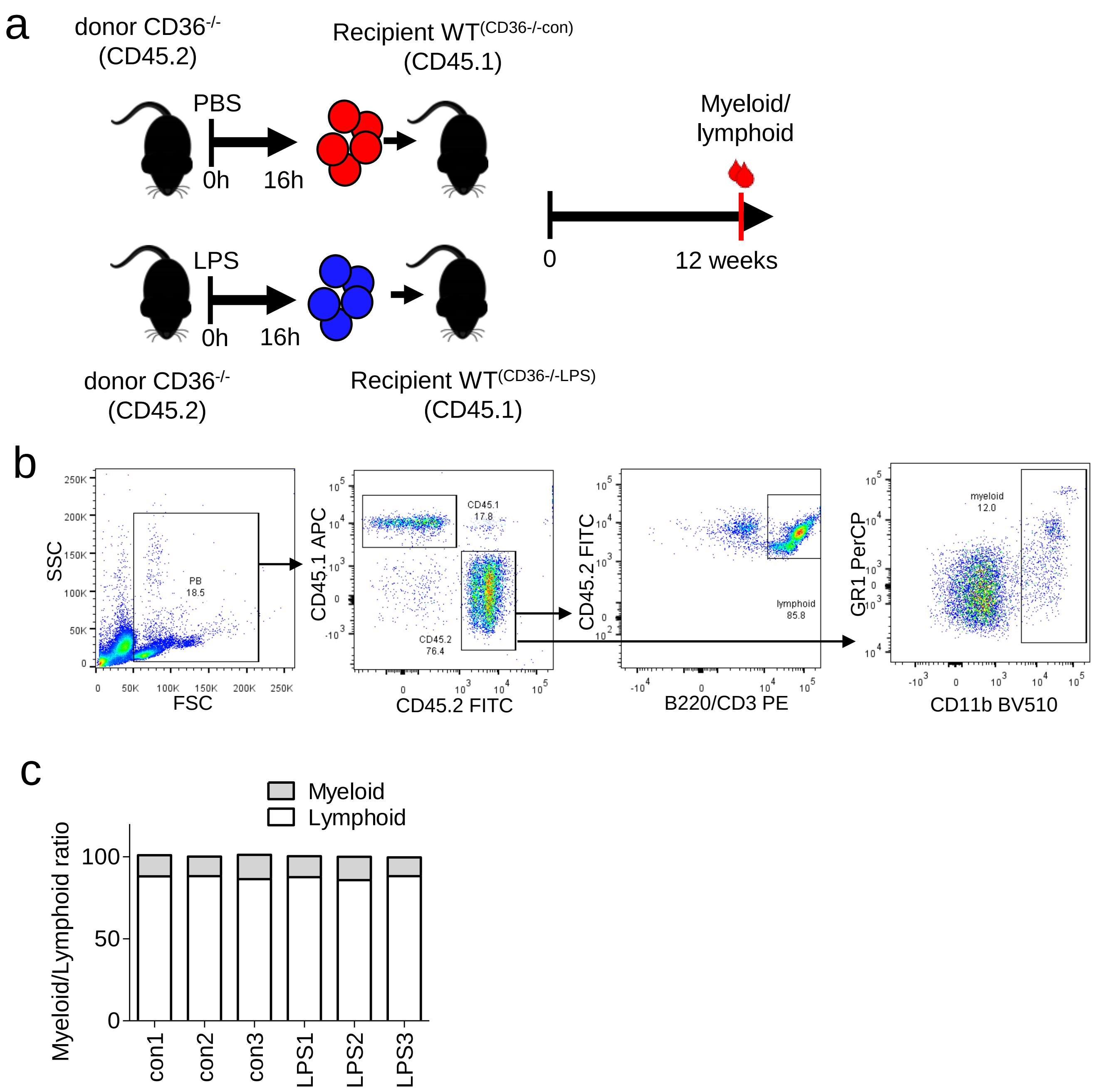

Supplementary Figure 8. Engraftment showing myeloid lymphoid ratios of LPS treated CD36$^{-/-}$ engrafted into WT animals. (A) Schematic of experimental design. CD36$^{-/-}$ CD45.2 mice were treated with control PBS or 1mg/kg LPS for 16 hours. The mice were scarified and the HSCs were isolated by FACS and transplanted into WT CD45.1 animals. 12 weeks post transplantation peripheral blood was analysed for CD45.2 expressing myeloid and lymphoid cells. (B) Gating strategy to isolate myeloid and lymphoid cell populations. (C) Percentage of CD45.2 expressing myeloid and lymphoid cells. n=3 mice in each group.

Supplementary figure 9

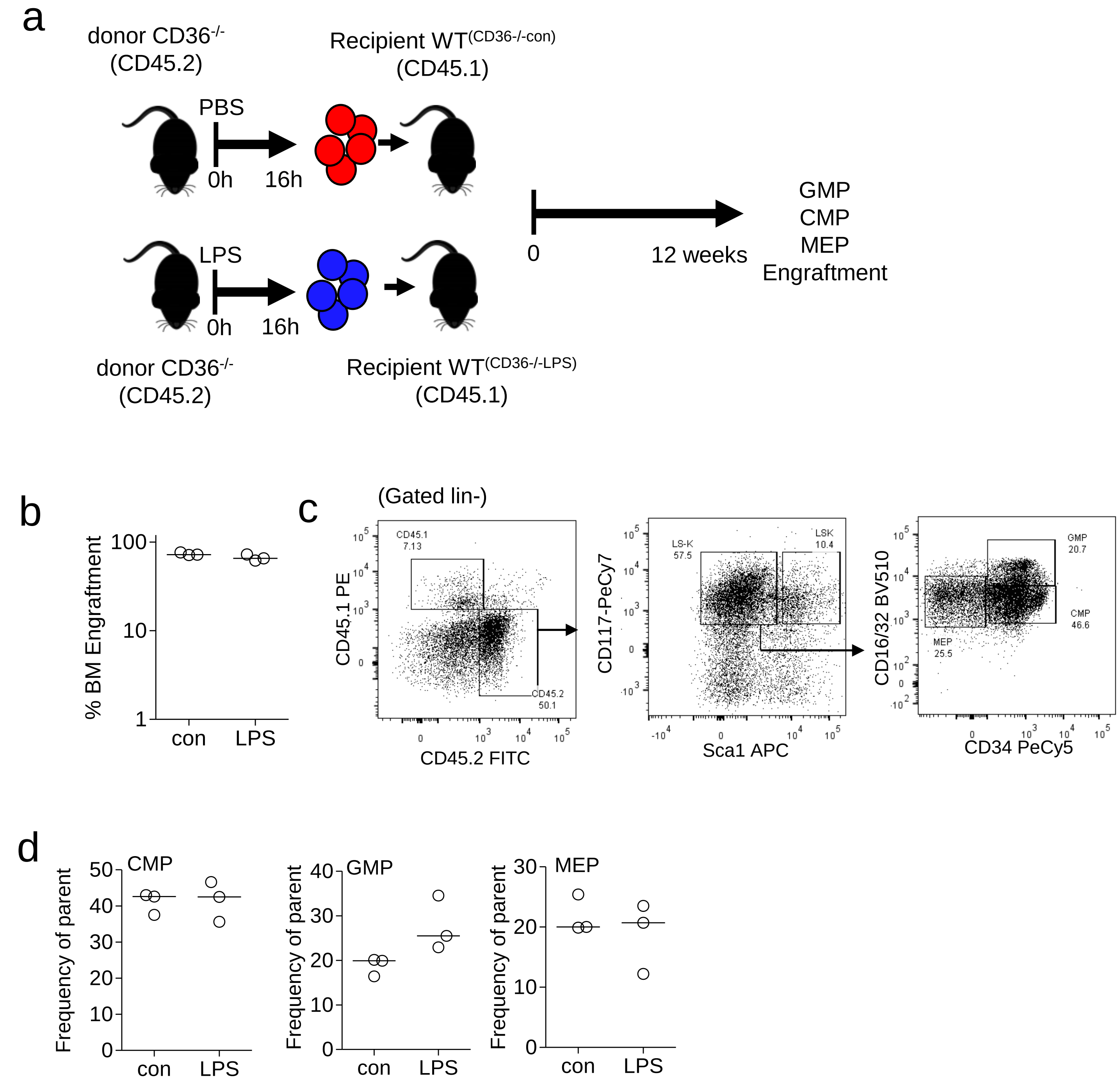

Supplementary Figure 9. Engraftment showing myeloid lymphoid ratios of LPS treated CD36$^{-/-}$ engrafted into WT animals. (A) Schematic of experimental design. CD36$^{-/-}$ CD45.2 mice were treated with control PBS or 1mg/kg LPS for 16 hours. The mice were scarified, and the HSCs were isolated by FACS and transplanted into WT CD45.1 animals. 12 weeks post transplantation animals were sacrificed, and the bone marrow extracted. (B) Percentage of CD45.2 engrafted cells in the bone marrow. (C) Gating strategy to isolate common myeloid progenitor (CMP), granulocyte macrophage progenitor (GMP) and megakaryocyte-erythrocyte progenitors (MEP). (D) Frequency of CD45.2 expressing CMP, GMP and MEP populations. n=3 mice in each group.

Supplementary Table 1

Gene Name	Forward Primer 5'-3'	Reverse Primer 5'-3'
Slc27a1	CAAGTACAATTGCACGGTAG	GTGAACTCCTCCCAGATG
Slc27a2	AAGAAGTGAATGTGTATGGC	GTTTTCTTTGATCTTGAGGGAG
Slc27a4	CTCAGCTATCTGTGAGATCC	GAGCTTATCGTTAAAACCCTTG
Slc27a5	CTTGTATGTGGGTGAAATCC	CACATAGTTCATTAAGCCCAC
Fabp1	AAATCAAACTCACCATCACC	GATTGTGTCTCCATTGAGTTC
Fabp3	AAACTCATCCTGACTCTCAC	AAAATGTCAGAGGGGAAAAC
Fabp4	GTAAATGGGGATTTGGTCAC	TATGATGCTCTTCACCTTCC
Fabp5	ATGAAAGAGCTAGGAGTAGG	TACAAGAGAACACAGTCGTC
CD36	CATTTGCAGGTCTATCTACG	CAATGTCTAGCACACCATAAG
Msr1	GCGGATCAAGATCACTATAAC	GGTGAAAGGTCTTTTAAGGAG
LDLR	CATCTTCTTCCCTATTGCAC	ATGCTGTTGATGTTCTTCAG
CPT1A	GGGAGGAATACATCTACCTG	GAAGACGAATAGGTTTGAGTTC

Supplementary Table 1. Oligonucleotides used for the detection of genes highlighted by RT-PCR
