# Supplementary material for: Free fatty-acid transport via CD36 drives β-oxidation-mediated hematopoietic stem cell response to infection
Source: Nat Commun. 2021 Dec 8;12:7130. doi: 10.1038/s41467-021-27460-9 (PMC8655073; doi:10.1038/s41467-021-27460-9)
Supplement: Supplementary file 3 — Reporting Summary [file 41467_2021_27460_MOESM3_ESM.pdf]

nature research

Corresponding author(s): Stuart Rushworth, Kristian Bowles, Naiara Beraza

Last updated by author(s): Sep 10, 2020

Reporting Summary

Nature Research wishes to improve the reproducibility of the work that we publish. This form provides structure for consistency and transparency in reporting. For further information on Nature Research policies, see our Editorial Policies and the Editorial Policy Checklist.

Statistics

For all statistical analyses, confirm that the following items are present in the figure legend, table legend, main text, or Methods section.

n/a	Confirmed	
☐	☒	The exact sample size (*n*) for each experimental group/condition, given as a discrete number and unit of measurement
☒	☐	A statement on whether measurements were taken from distinct samples or whether the same sample was measured repeatedly
☐	☒	The statistical test(s) used AND whether they are one- or two-sided<br>*Only common tests should be described solely by name; describe more complex techniques in the Methods section.*
☒	☐	A description of all covariates tested
☒	☐	A description of any assumptions or corrections, such as tests of normality and adjustment for multiple comparisons
☐	☒	A full description of the statistical parameters including central tendency (e.g. means) or other basic estimates (e.g. regression coefficient) AND variation (e.g. standard deviation) or associated estimates of uncertainty (e.g. confidence intervals)
☐	☒	For null hypothesis testing, the test statistic (e.g. *F*, *t*, *r*) with confidence intervals, effect sizes, degrees of freedom and *P* value noted<br>*Give P values as exact values whenever suitable.*
☒	☐	For Bayesian analysis, information on the choice of priors and Markov chain Monte Carlo settings
☒	☐	For hierarchical and complex designs, identification of the appropriate level for tests and full reporting of outcomes
☒	☐	Estimates of effect sizes (e.g. Cohen's *d*, Pearson's *r*), indicating how they were calculated

Our web collection on statistics for biologists contains articles on many of the points above.

Software and code

Policy information about availability of computer code

Data collection	BD FACS Diva software 8.0.1, Wave 2.6.1, Zenbio for image acquisition. Serum samples were analsyed in a Randox RX Daytona Analyzer.
Data analysis	Graphpad Prism 7, BD FlowJo 10.7.0, Wave 2.6.1, Fiji ImageJ 2.0.0.

For manuscripts utilizing custom algorithms or software that are central to the research but not yet described in published literature, software must be made available to editors and reviewers. We strongly encourage code deposition in a community repository (e.g. GitHub). See the Nature Research guidelines for submitting code & software for further information.

Data

Policy information about availability of data

All manuscripts must include a data availability statement. This statement should provide the following information, where applicable:

- Accession codes, unique identifiers, or web links for publicly available datasets
- A list of figures that have associated raw data
- A description of any restrictions on data availability

The authors declare that the data supporting the findings of this study are available within the paper and supplementary information files. Individual data points are highlighted on the plots provided. Source data file is also included.

Field-specific reporting

Please select the one below that is the best fit for your research. If you are not sure, read the appropriate sections before making your selection.

☒ Life sciences ☐ Behavioural & social sciences ☐ Ecological, evolutionary & environmental sciences

For a reference copy of the document with all sections, see nature.com/documents/nr-reporting-summary-flat.pdf

Life sciences study design

All studies must disclose on these points even when the disclosure is negative.

Sample size	Based on our previous work published in Blood (Marlein 2017) and PNAS (Mistry 2019) we performed power calculations to determine sample sizes for this study
Data exclusions	Criteria for excluding samples or data for in vivo experiments include failure to meet quality control standards, such as insufficient sample volume or animals may be dropped from the experiment if the reach endpoints before the pre-determined one. Due to COVID-19 lockdown experiment located in Figure 3G-J we lost 2 animals in the CPT1A KD arm. We also lost 2 animals in Figure 3A,B and D in the salmonella arm
Replication	All experiments are represented by multiple biological replicates, as indicated in the legends. We confirm all replication attempts were successful. The ex vivo assays were performed in triplicate. No in vitro assays were performed.
Randomization	Mice were randomly allocated treatment groups based on number (eg even number =control odd number = test group).
Blinding	The investigators were not blinded for tissue harvest or processing due to the risk of confusion in handling. The investigators were not blinded during acquisition or analysis of flow cytometry data, but the same gating were applied to all samples collected in a given experiment.

Reporting for specific materials, systems and methods

We require information from authors about some types of materials, experimental systems and methods used in many studies. Here, indicate whether each material, system or method listed is relevant to your study. If you are not sure if a list item applies to your research, read the appropriate section before selecting a response.

Materials & experimental systems

n/a	Involved in the study	
☐	☒	Antibodies
☒	☐	Eukaryotic cell lines
☒	☐	Palaeontology and archaeology
☐	☒	Animals and other organisms
☒	☐	Human research participants
☒	☐	Clinical data
☒	☐	Dual use research of concern

Methods

n/a	Involved in the study	
☒	☐	ChIP-seq
☐	☒	Flow cytometry
☒	☐	MRI-based neuroimaging

Antibodies

Antibodies used	Flow cytometry antibodies were purchased from Biolegend, Miltenyi Biotec and ThermoFisher. Lineage cocktail Pacific Blue anti-mouse Biolegend Catalogue number:133310 Lot number: B289722 (dilution 1:50). Components include anti-mouse CD3, clone 17A2 (dilution 1:100); anti-mouse Ly-6G/Ly-6C, clone RB6-8C5 (dilution 1:100); anti-mouse CD11b, clone M1/70 (dilution 1:100); anti-mouse CD45R/B220, clone RA3-6B2 (dilution 1:50); anti-mouse TER-119/Erythroid cells, clone Ter-119 (dilution 1:50). CD117 Pe-Vio 770 anti-mouse Miltenyi Biotec Catalogue number:130-111- 695 Lot number: 5200705289 Clone: REA791 (dilution 1:50). Sca-1 APC mouse Miltenyi Biotec Catalogue number:130-123-848 Lot: 5191212189 Clone: REA422 (dilution 1:50). CD150 (SLAM) Brilliant Violet 510 anti-mouse Biolegend Catalogue number:115929 Lot number: B264811 Clone: TC15-12F12.2 (dilution 1:50). CD48 APC-Cyanine 7 Anti-mouse Biolegend Catalogue number: 103432 Lot number: B271042 Clone: HM48-1 (dilution 1:50). CD34 PerCP/Cyanine 5.5 Anti-mouse Biolegend Catalogue number: 128608 Lot number: B271491 Clone: HM34 (dilution 1:50). CD36 VioBright 515 mouse Miltenyi Biotec Catalogue number: 130-122-094 Lot number: 520020623 Clone: REA1184 (dilution 1:50). Anti Ki-67 FITC human and mouse Miltenyi Biotec Catalogue number: 130-100-339 Clone: REA183 Lot number: 5171102132 (dilution 1:100). CD45.1 Antibody, anti-mouse, APC Miltenyi Biotec Catalogue number: 130-102-470 Clone: A20 (dilution 1:200). CD45.1 Antibody, anti-mouse, PE Miltenyi Biotec Catalogue number: 130-102-499 Clone: A20 (dilution 1:200). CD45.2 Antibody, anti-mouse, FITC Miltenyi Biotec Catalogue number: 130-102-997 Clone: 104-2 (dilution 1:200). Anti-FABP3 clone and catalogue number PA5-13461, ThermoFisher (dilution 1:50).
Validation	All antibodies used were purchased from commercial companies and have been validated by the companies. statements for validation of antibodies can be found from Biolegend https://www.biolegend.com/en-us/quality/quality-control Antibodies purchased from Miltenyi Biotech https://www.miltenyibiotec.com/GB-en/products/macs-antibodies/antibody-

validation.html?countryRedirected=1#gref. For antbodies from ThermoFisher https://www.thermofisher.com/us/en/home/life-science/antibodies/invitrogen-antibody-validation.html

Animals and other organisms

Policy information about studies involving animals; ARRIVE guidelines recommended for reporting animal research

Laboratory animals	C57BL/6J mice (CD45.2), were purchased (Charles River Massachusetts, United States). B6.SJL-PtprcaPep3b/BoyJ (CD45.1) (PepCboy) mice and B6.129S1-Cd36tm1Mfe/J (CD36-/-) were purchased from The Jackson Laboratory (Bar Harbour, ME, USA). Animals were housed in a specific pathogen-free facility. Mice used were at age 8-12 weeks of age and both genders were used for experiments with the exception of mice that were used for transplantation which 3-4-week-old mice were used. Mice were individually ventilated and housed under specific pathogen-free conditions in a 12/12-hour light/dark cycle with food and water provided ad libitum in accordance with the Animal (Scientific Procedures) Act, 1986 (UK). The room temperature for mice was 22°C and the relative humidity is kept at between 45 to 65%.
Wild animals	Study did not involve wild animals
Field-collected samples	Study did not involve field collected samples
Ethics oversight	All animal work used in this study were carried out in accordance with regulations set by the UK Home Office and the Animal Scientific Procedures Act 1986.

Note that full information on the approval of the study protocol must also be provided in the manuscript.

Flow Cytometry

Plots

Confirm that:

☒ The axis labels state the marker and fluorochrome used (e.g. CD4-FITC).

☒ The axis scales are clearly visible. Include numbers along axes only for bottom left plot of group (a 'group' is an analysis of identical markers).

☒ All plots are contour plots with outliers or pseudocolor plots.

☒ A numerical value for number of cells or percentage (with statistics) is provided.

Methodology

Sample preparation	Antibody cocktails were prepared in MACs buffer and incubated with bone marrow cells for at least 30 min at 4°C. Experiments using 4- difluoro-1,3,5,7,8-pentamethyl-4-bora-3a,4a-diaza-s-indacene (BODIPY 493/503), the cells were incubated with BODIPY 493/503 (1µM, Invitrogen) at room temperature for 20min, washed twice in 1 X PBS and centrifuged at 1200rpm for 5 minutes before staining with antibody cocktail. For experiments using Ki67 the bone marrow cells were incubated with antibody cocktail prior to fixing and permeabilization using the FIX & PERM™ Cell Permeabilization Kit (ThermoFisher, Waltham, MA, USA) as per manufacturer's instructions. The cells were then stained with Anti-Ki-67-FITC, human and mouse (Miltenyi Biotec, Germany) for 20 min centrifuged at 1200rpm for 5 minutes before resuspending in PBS. For flow cytometric cell sorting of bone marrow cell populations pellet was re-suspended in antibody mix and cells were sorted directly into lysis buffer.
Instrument	BD FACSCanto II and BD FACSMelody (BD Biosciences)
Software	BD FACS Diva software 8.0.1 and FlowJo 10.7.0 (BD Biosciences)
Cell population abundance	Abundance of Sca-1+ ckit+ (LSK) cells in lineage negative gate ranged from 3-15% depending on stimulus. Abundance of CD150+ CD48- (HSC) in LSK gate ranged from 4-9% depending on stimulus
Gating strategy	Bone marrow cells were initiallly gated on FSC/SSC. The gate used excluded red cells and debris. We then used flourescence minus one to set the positive and negative gates

☒ Tick this box to confirm that a figure exemplifying the gating strategy is provided in the Supplementary Information.
